# Plasticity of parental CENH3 incorporation into the centromeres in wheat × barley F1 hybrids

**DOI:** 10.3389/fpls.2024.1324817

**Published:** 2024-01-19

**Authors:** Edit Mihók, Dávid Polgári, Andrea Lenykó-Thegze, Diána Makai, Attila Fábián, Mohammad Ali, András Kis, Adél Sepsi, László Sági

**Affiliations:** ^1^ Centre for Agricultural Research, Hungarian Research Network, Martonvásár, Hungary; ^2^ Doctoral School of Plant Sciences, Hungarian University of Agriculture and Life Sciences, Gödöllő, Hungary; ^3^ Institute of Genetics and Biotechnology, Hungarian University of Agriculture and Life Sciences, Gödöllő, Hungary; ^4^ Agribiotechnology and Precision Breeding for Food Security National Laboratory, Plant Biotechnology Section, Centre for Agricultural Research, Martonvásár, Hungary

**Keywords:** centromeric DNA, chromosome elimination, *Hordeum vulgare*, interspecific hybridization, *Triticum aestivum*

## Abstract

Incorporating the centromere-specific histone H3 protein CENH3 into the centromeric nucleosomes is indispensable for accurate centromere function and balanced chromosome segregation in most eukaryotes, including higher plants. In the cell nuclei of interspecific hybrids, divergent centromeric DNAs cohabit and lead the corresponding parental chromosomes through the mitotic and meiotic cell divisions. Depending on the transmission of the parental chromosomes carrying the CENH3-encoding genes, CENH3 proteins from one or both parents may be present in these hybrids. The incorporation of parental CENH3 proteins into the divergent centromeres and their role in the chromosome elimination process in interspecific hybrids is still poorly understood. Here, we produced wheat × barley F1 hybrids that carried different combinations of barley chromosomes with genes encoding for either one (αCENH3) or both barley CENH3 protein variants (α– and βCENH3). We generated specific antibodies distinguishing between the wheat CENH3 proteins and barley αCENH3 and applied them together with FISH probes to detect the precise pattern of parental CENH3 deposition into the wheat and barley centromeric nucleosomes. Analysis of somatic and meiotic nuclei of the wheat × barley hybrids revealed the plasticity of the maternal (wheat) CENH3 proteins to become incorporated into the paternal (barley) centromeric nucleosomes. However, no evidence for paternal CENH3 plasticity was detected in this study. The significance of the unilateral centromere plasticity and possible patterns of CENH3 incorporation into centromeres in interspecific hybrids are discussed.

## Introduction

Combining the genomes of distantly related species via interspecific (or wide-cross) hybridisation is a key strategy to increase the genetic diversity of cultivated plants and enhance crop resilience under the changing climate. Agronomic improvement of bread wheat (*Triticum aestivum* L.) depends on interspecific hybridisation because thousands of years of inbreeding have narrowed down its genetic diversity (Levy and Feldman, 2022). Fertilisation via artificial crossing between two related cereal species generally results in male-sterile F1 hybrids that carry the haploid chromosome set of both parents. The reconstruction of the original diploid chromosome sets is required to restore fertility, which can be achieved via spontaneous (Rimpau, 1891; Karpechenko, 1928) or chemically induced genome duplication (Györffy and Melchers, 1938; Sears, 1939; Nemeth et al., 2015), or successive backcrossing and selfing (Harlan and Pope, 1922; Briggs, 1930; Florell, 1931; O'Mara, 1940; Feldman, 1965; Islam et al., 1981). This way, a wide range of new traits or trait combinations were introduced into the fully reconstructed wheat genome (Sears, 1956; Kimber, 1967; Riley et al., 1968; Friebe et al., 1996; Kumar et al., 2017). Today, as wheat production faces challenges imposed by the climate crisis, considerable efforts are underway to transfer and unlock the full genetic potential of cultivated and wild relatives into wheat (King et al., 2017; Türkösi et al., 2018; Reynolds et al., 2021; Leigh et al., 2022). Incorporating the vast biodiversity accumulated across related species would generate a readily useable gene pool for wheat breeding. The combination of the genomes of wheat and barley is still limited due to numerous challenges met during and after fertilisation. Random and partial or complete elimination of barley chromosomes (Finch and Bennett, 1982; Polgári et al., 2019) is an important bottleneck to the transfer of barley traits into wheat. Despite its prevalence, the mechanisms behind the elimination of barley chromosomes from the wheat background are poorly understood. The specialised chromosomal regions ensuring chromosome movement at mitosis and meiosis are the centromeres. Centromeres are responsible for spindle attachment and accurate chromosome segregation during cell division (Henikoff and Malik, 2002; Westhorpe and Straight, 2013) and their inactivity was proposed as one of the triggering factors of selective chromosome elimination (Sanei et al., 2011). Centromere function across most eukaryotes, including higher plants, is determined epigenetically by the substitution of the canonical H3 histone protein to the centromeric H3 histone (CENH3) protein in the centromeric nucleosomes (Houben and Schubert, 2003; Lermontova et al., 2015; Liu et al., 2015). Although CENH3 proteins show remarkable functional conservation, their amino acid sequences demonstrate rapid evolutionary changes. The C-terminal histone-fold domain, oriented towards the globular core of the nucleosome, shows a slower evolution rate while the N-terminal tail domain protruding from the nucleosome and interacting with the centromeric DNA is highly variable (Talbert et al., 2002; Ravi et al., 2010; Maheshwari et al., 2015).

In barley, two paralogous *CENH3* genes encode the α– and βCENH3 protein variants located on chromosomes 1H and 6H, respectively (Sanei et al., 2011). The two CENH3 variants were also identified in wheat with each of them being encoded by three copies of genes located on the homoeologous group 1 chromosomes of the A, B, and D sub-genomes (Yuan et al., 2015). Phylogenetic analysis of CENH3 protein sequences within the *Triticeae* tribe divided the α– and βCENH3 proteins into two distinct groups: the αCENH3 proteins of wheat and barley cluster together while the βCENH3 proteins of wheat cluster with that of barley (Yuan et al., 2015). Similarities between the different CENH3 variants of wheat and barley are only partially reflected in the structure of their cognate centromeric DNAs that contain conserved motifs interspersed with highly divergent centromeric DNA sequences (Hudakova et al., 2001; Li et al., 2013), indicative of the accelerated evolutionary rate within the centromeric regions. The plant centromeric DNA typically consists of long arrays of short satellite repeat motifs and retroelements which are intermingled with a few low copy-number sequences including actively transcribed genes (Cheng et al., 2002; Zhong et al., 2002; Heslop-Harrison et al., 2003; Nagaki et al., 2011; Qi et al., 2013; Naish et al., 2021). Accordingly, the DNA of barley centromeres is mainly composed of the *gypsy*-like Long Terminal Repeat (LTR) retrotransposon *cereba* (Aragón-Alcaide et al., 1996; Jiang et al., 1996; Presting et al., 1998) and the short G+C-rich centromeric satellite (AGGGAG)n sequence (Hudakova et al., 2001). Both *cereba* and the G+C-rich centromeric satellite bind CENH3 indicating that they are constituents of the active centromere (Houben et al., 2007). In wheat, the DNA component of the active centromeres is mainly composed of the centromere-specific retrotransposon (CRW), which is orthologous to the barley *cereba* sequence, and arrays of satellite repeats (Zhang et al., 2004; Li et al., 2013; Zhao et al., 2023). Some of the short centromeric satellite motifs have, however, lost the capacity to bind CENH3 (Kishii et al., 2001; Li et al., 2013; Su et al., 2019). Another major element of the wheat core centromere is *Quinta*, a high-copy LTR retrotransposon completely absent in barley, which binds CENH3 at a higher affinity when compared to CRW (Li et al., 2013; Zhao et al., 2019).

In the present work, we evaluated cross-species CENH3 incorporation into the centromeres of newly developed wheat × barley F1 hybrid plants to understand centromere plasticity in loading CENH3 proteins from distantly related parental species and its possible role in uniparental chromosome elimination. Two wheat × barley F1 hybrid plants carrying different combinations of wheat and barley chromosomes were selected for cytological examinations. We developed antibodies selectively recognising the wheat and barley CENH3 proteins and verified their loading into the centromeric nucleosomes of each parent species. Parental centromeric DNAs were then identified within single somatic and meiotic cell nuclei of the F1 hybrid plants and the incorporation of species-specific CENH3 proteins was monitored by immunoFISH. Our study gives an insight into the compatibility of centromeres, derived from distantly related parental species but co-located in the same cell nucleus, to incorporate same-species or cross-species CENH3 proteins and maintain chromosome stability.

## Materials and methods

### Plant materials

Wheat × barley F1 hybrids were produced by crossing the doubled haploid ‘M1’ wheat (derived from the spring landrace ‘Sichuan’; Polgári et al., 2014) with the two-row spring barley cultivar ‘Golden Promise’. The parental lines were grown in reach-in growth chambers (Conviron, Winnipeg, Canada) in the Phytotron Facility of the Centre for Agricultural Research (Martonvásár, Hungary) at a constant 18°C temperature under a 16-h photoperiod. Wheat florets were emasculated and pollinated with barley as described by Polgári et al. (2014). Embryos were rescued on the 14^th^ day after pollination and plants were regenerated on the N6D medium (Chu et al., 1975). F1 plantlets were subjected to a six-week vernalisation period (+4°C, 12 h photoperiod) after which they were potted and incubated in growth cabinets (MLR-352-PE, PHCbi, Panasonic Corporation, Kadoma, Japan) at 21°C/18°C (day/night) and 16 h photoperiod.

### Molecular marker analysis

Total DNA was isolated from young leaves by a direct extraction method. Briefly, an approx. 5×5 mm leaf section was homogenised in a 1.5 mL Eppendorf tube including a stainless-steel bead (D=3 mm, Qiagen, Venlo, the Netherlands) and 100 µL of Extraction solution (E7526-24ML, Sigma-Aldrich, St Louis, MO, USA). Homogenisation was performed in a mixer mill (Bullet Blender Storm Pro, Next Advance, Troy, NY, USA). The homogenate was incubated at 95°C for 15 min, cooled on ice (1 min), and diluted with 100 µL of Dilution solution (Sigma-Aldrich, D5688-12ML). After vortexing, the samples were centrifuged at 18,000 × g for 1 min at room temperature and the supernatant was stored at –20°C until use.

Barley chromosome-specific (1H-7H) primer pairs as listed in Polgári et al. (2019) were used to identify individual barley chromosomes in the wheat × barley F1 hybrids. PCR reactions were carried out in a final volume of 20 µL containing 1 µL of DNA, 4 µL of 5X Phusion HF Buffer (Thermo Scientific, Waltham, MA, USA, F538), 0.2 µL of Phusion Hot Start II High-Fidelity DNA Polymerase (2 U/µL, Thermo Scientific, F549), 0.5 µM of each of the forward and reverse primers, 4 µM of dNTPs (Thermo Scientific, R1121), adjusted with sterile water. The PCR cycles for chromosomes 1H, 2H, and 3H involved an initial 3-min denaturation at 98°C followed by 34 cycles of 98°C for 10 s, 65°C for 15 s, and 72°C for 25 s, and a final extension step at 72°C of 10 min. For the chromosomes 4H, 5H, 6H, and 7H, the annealing temperature was modified to 61°C. PCR reactions were performed in a Mastercycler nexus gradient thermal cycler (Eppendorf, Hamburg, Germany). The amplification products were analysed by gel electrophoresis in a 1.2% (w/v) agarose gel stained with ethidium bromide (0.5 µg/mL). Gel images were captured in the ChemiDoc MP Imaging System (Bio-Rad Laboratories, Hercules, CA, USA).

### Simultaneous GISH-FISH

Somatic nuclei and chromosome spreads were prepared from fixed (ethanol: acetic acid, 3:1) root tips using the squash method (Kruppa et al., 2013). The GISH probe was obtained by labelling total DNA from ‘Golden Promise’ barley with nick-translation (AF594 NT Labeling Kit, PP-305L-AF594; Jena Bioscience, Jena, Germany). To obtain the FISH probe, the DNA sequence covering the barley 5S rDNA coding and noncoding flanking regions (Fukui et al., 1994) was amplified by PCR and labelled with an AF488 NT Labeling Kit (Jena Bioscience, PP-305L-AF488). *In situ* hybridisation was performed according to Lenykó-Thegze et al. (2021) with minor adjustments. The probe mixture contained 54% (v/v) of deionised formamide (Sigma-Aldrich, F9037), 2.4% (w/v) dextran sulphate (Sigma-Aldrich, 67578) diluted in saline sodium citrate buffer (2X SSC: 0.3 M NaCl, 30 mM trisodium citrate dihydrate, pH 7.0). Fourty to eighty ng of each labelled probe per slide was supplemented with 1500 ng of unlabelled wheat DNA. The probe mixture was denatured at 85°C for 8.5 min and immediately chilled on ice. When the final volume of 22 µL of probe mix was applied, the slides were again denatured at 75°C for 3 min. Hybridisation was allowed overnight at 37°C.

### Design and production of species- and variant-specific anti-CENH3 antibodies

Short (11-14 aa) peptides were designed for raising polyclonal antibodies to recognise the α– and βCENH3 variants of wheat and barley by using the multiple sequence alignment tool of Clustal Omega (Sievers et al., 2011; https://www.ebi.ac.uk/Tools/msa/clustalo/). The CENH3 amino acid sequence alignments were based on the following UniProtKB (https://www.uniprot.org/) entries: wheat αCENH3 – A-genome: I3NV45, B-genome: I3NV43, D-genome: I3NV44 (Yuan et al., 2015), barley αCENH3: G1APU2 (Sanei et al., 2011); wheat βCENH3 – A-genome: A0A3B5Y4B2, B-genome: A0A3B5Z1Q8, *Aegilops tauschii* βCENH3 D-genome: A0A0G3YL56 (Yuan et al., 2015), barley βCENH3: G1APU3 (Sanei et al., 2011). Peptides having multiple matches with similar properties were excluded by the EMBOSS Matcher tool (EMBL-EBI) to avoid potential unwanted antibody cross-linking. All four peptide sequences were selected from the variable N-terminal tail domain of the wheat and barley CENH3 amino acid sequences. The 3D structural models of the species-specific CENH3 protein variants (**Supplementary Figure 3**) were created with the AlphaFold Monomer v2.0 pipeline (Jumper et al., 2021) and can be downloaded from the AlphaFold Protein Structure Database (Varadi et al., 2022; https://alphafold.ebi.ac.uk/).

The synthetised peptides were conjugated to keyhole limpet hemocyanin or bovine serum albumin proteins as carriers and injected into live animals for immunisation according to a standard 90-day protocol at DC BioScience Ltd. (Dundee, UK). The antibodies were raised against the following peptides of the α– and βCENH3 variants of wheat and barley: a guinea pig anti-wheat αCENH3 antibody (Wα, peptide sequence KKQLGPRPAQR), a rat anti-wheat βCENH3 antibody (Wβ, peptide sequence KRLRFELSPRWRP), a sheep anti-barley αCENH3 antibody (Bα, peptide sequence: KKIGSASSPSA) and a rabbit anti-barley βCENH3 antibody (Bβ, peptide sequence CSKSEPQSQPKKKE).

### Immunolabelling

Fixation and preparation of nuclei were carried out according to Makai et al. (2023). Briefly, root tips were fixed in 4% paraformaldehyde (PFA, diluted in 1X PBS from isotonic 16% (w/v) Paraformaldehyde Solution; Thermo Scientific, 28908) 0.5% (v/v) Igepal CA-630 (Sigma-Aldrich, 18896) for 30 min, with the first 5 min involving vacuum infiltration. The fixed root tips were homogenised in LB01 lysis buffer [15 mM Tris-HCl, 2 mM Na_2_EDTA, 0.5 mM spermine, 80 mM KCl, 20 mM NaCl, and 0.1% (v/v) Triton X-100 (Sigma-Aldrich, T8787), pH 8.0; Doležel et al., 1989] in a 2 mL KIMBLE Dounce tissue grinder set (Sigma-Aldrich, D8938). The cell suspension was filtered through a 70-µm and 40-µm cell strainer (pluriStrainer Mini 70 µm, 43-10070-40 and pluriStrainer Mini 40 µm, 43-10040-40; pluriSelect Life Science, Leipzig, Germany) and centrifuged at 2,000 × g for 5 min at 4°C. Five to eight µL of cell suspension were pipetted per adhesion microscope slides (Erpedia Superfrost Plus Adhesion Microscope Slides; Menzel-Gläser, Braunschweig, Germany). Immunolabelling was carried out as described by Sepsi et al. (2017) with minor modifications. Primary antibodies were diluted at a ratio of 1:50-100 in 1X TNB blocking buffer [0.1 M Tris–HCl, pH 7.5, 0.15 M NaCl, and 0.5% (w/v) Blocking Reagent, Roche Diagnostics, Basel, Switzerland, 11096176001] containing 0.3 M glycine (Sigma-Aldrich, G8898), 0.2% Triton X-100, 0.2% Igepal, and 0.025% (w/v) saponin (Sigma-Aldrich, 47036). The following secondary antibodies (all labelled with abberior STAR RED, Abberior GmbH, Göttingen, Germany) were used: goat anti-guinea pig IgG (STRED-1006), goat anti-rat IgG (STRED-1007), donkey anti-sheep (STRED-1056), goat anti-rabbit IgG (STRED-1002).

### ImmunoFISH

Somatic nuclei were fixed and prepared by the method described for the immunolabelling procedure (see above). Anthers were fixed in 4% paraformaldehyde with 0.5% Igepal CA-630 for 15 min, the first 5 min with vacuum infiltration. Pollen mother cells were slide-mounted with a pair of fine tungsten needles by squeezing into a drop of 1X PBS-0.5% Igepal. The specimens were allowed to air dry and were then snap-frozen on dry ice. FISH probes were obtained by PCR amplification of a 576-bp fragment from the integrase region of the polyprotein gene of the wheat centromeric retrotransposon (CRW) by using the primers 5’-GTTTGTCCATCAGTTTGG-3’ and 5’-GTTTGTCCATCAGTTTGG-3’ and by amplification of the barley centromere-specific G+C-rich satellite sequence (Hudakova et al., 2001). The amplified CRW and G+C-rich satellite sequences were labelled by nick-translation (DIG-Nick Translation Mix, Roche, 11745816910 and BioNick DNA Labeling System Cat. no. 18247015, Invitrogen, Carlsbad, CA, USA). Digoxigenin and biotin signals were detected by Anti-Digoxigenin-Rhodamine (Roche, 11207750910) and Streptavidin-FITC (Sigma-Aldrich, S3762), respectively. The immunoFISH procedure was carried out as described by Sepsi et al. (2018). The slides were mounted in 18 µL of Vectashield Antifade Mounting Medium with DAPI (H-1200; Vector Laboratories, Burlingame, CA, USA).

### Confocal microscopy

The detection of fluorescence signals was performed by an SP8 TCS confocal laser scanning microscope (Leica Microsystems GmbH, Wetzlar, Germany). The DNA stain DAPI was excited at 405 nm and detected between 410-470 nm. The detection settings for the various labelled secondary antibodies used in the present work were as follows: Alexa Fluor 488 was excited at 488 nm and detected between 490-560 nm; Alexa Fluor 594 was excited at 561 nm, detected between 600-660 nm; abberior STAR RED was excited at 633 nm and detected between 650-700 nm. A series of confocal images (“*z* stacks”) with a lateral (*x* and *y*) resolution of 45 nm and an axial (*z*) resolution of 200 nm were acquired by an HC PL APO CS2 63×/1.40 oil immersion objective (Leica Microsystems). Image stack deconvolution was performed using the Huygens Essential software v18.04 (Scientific Volume Imaging, Hilversum, the Netherlands). No further manipulation was performed on the images.

## Results

### Molecular and cytological characterisation of wheat × barley F1 hybrids

**Figure 1:**
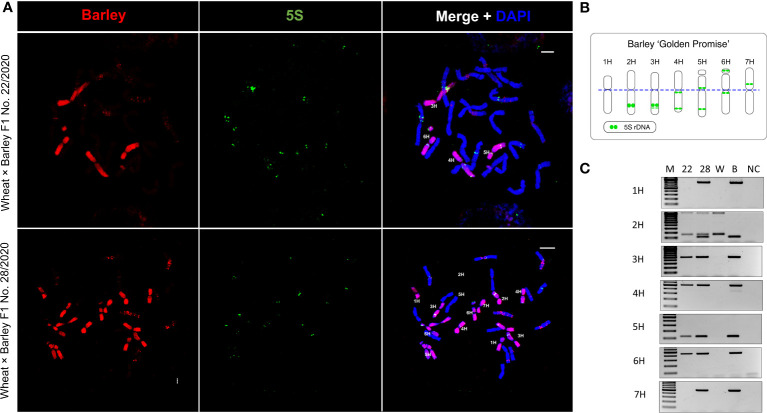
Molecular cytological (GISH-FISH) characterisation of the two wheat × barley primary (F1) hybrids studied. **(A)** The barley chromosome complement is visualised by GISH (labelled with red). Individual barley chromosomes are identified by FISH using a 5S rDNA-specific probe (green). The chromatin is counterstained with DAPI (blue on merge). Bars = 5 µm (upper panel) and 10 µm (lower panel). **(B)** Schematic karyogramme of barley ‘Golden Promise’ according to 5S rDNA-specific FISH signal distribution. **(C)** Identification of individual barley chromosomes (1H-7H) in the two hybrids with chromosome-specific PCR markers. M, size marker (GeneRuler 100 bp Plus, Thermo Scientific); 22 and 28, the two F1 hybrids; W and B, wheat and barley parental controls, respectively; NC, non-template control.

To analyse the mechanism of CENH3 incorporation in interspecific hybrids, two wheat × barley F1 hybrids were selected by initial screening with chromosome-specific PCR markers (**Figure 1**). A partial hybrid (No. 22/2020) that carried only four of the barley chromosomes, lacking the 1H but containing the 6H chromosome (αCENH3^-^, βCENH3^+^; **Figure 1**), was selected to examine whether the wheat αCENH3 can functionally compensate for the missing barley αCENH3 variant by loading into the barley centromeres. A full hybrid (No. 28/2020) carrying all seven chromosomes of barley (αCENH3^+^, βCENH3^+^, **Figure 1**) was chosen to test the incorporation of wheat CENH3, detected by antibodies specifically designed to the wheat αCENH3 and βCENH3 variants, into the barley centromeric nucleosome in the presence of both barley CENH3 variants and *vice versa*.

Cytological examination of root-tip cells (n=75) of the partial hybrid No. 22/2020 by simultaneous GISH-FISH using total barley DNA and 5S rDNA probes revealed 21 wheat chromosomes and four barley chromosomes in the somatic nuclei of this hybrid. The 5S rDNA FISH probe identified the barley chromosomes as 3H, 4H, 5H, and 6H (**Figures 1A, B**), confirming the absence of the 1H chromosome and thus that of the barley αCENH3-encoding gene. *In situ* hybridisation in root-tip cells (n=48) of the full hybrid No. 28/2020 unexpectedly detected 14 barley chromosomes suggesting the duplication of the paternal genome. A varying number of wheat chromosomes were observed in addition to the full barley chromosome set. In the majority (79%) of the cells analysed, the number of wheat chromosomes ranged from 14 to 20 (**Figure 1A**: lower panel) indicating their mitotic instability in this hybrid. In a subset (21%) of the mitotic nuclei analysed, only the chromosomes originating from the barley parent were retained indicating the progressive elimination of wheat chromosomes (**Supplementary Figure 1**).

### Species-specific immunolabelling of wheat and barley CENH3

**Figure 2:**
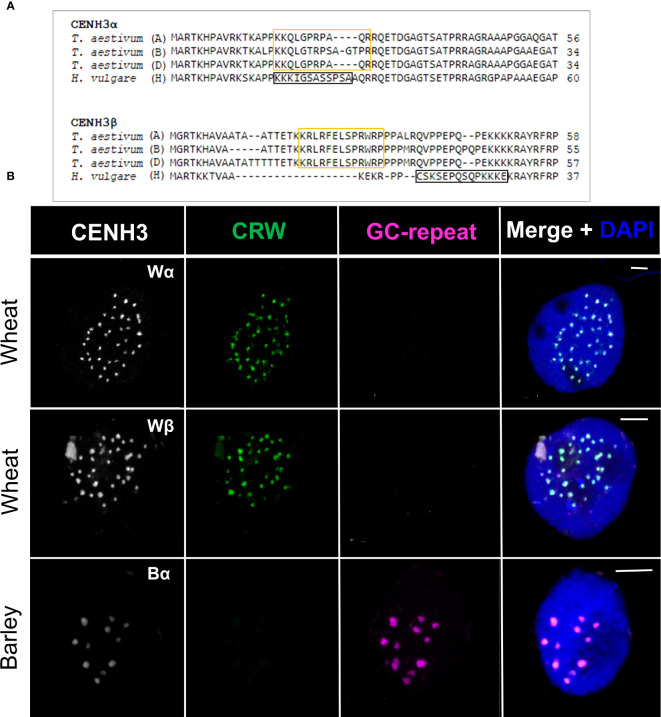
Design and evaluation of four anti-CENH3 antibodies. **(A)** Selection of target epitope peptides of the wheat and barley CENH3 proteins. Amino acid sequences were compared and aligned by Clustal Omega (www.ebi.ac.uk/Tools/msa/clustalo/). The positions used for the synthesis of the target peptides in wheat α– and βCENH3 are boxed in yellow. The peptide sequences used to produce the anti-barley α– and βCENH3 antibodies are boxed in black. **(B)** Centromere-specificity of anti-CENH3 antibodies in wheat and barley interphase nuclei by co-localisation with centromere-specific FISH probes. Active centromeres are immunolabelled with anti-CENH3 antibodies (white) while the centromeric retrotransposon of wheat (CRW, green) and the barley centromere-specific G+C-rich repeats (magenta) are detected by FISH. The chromatin is counterstained with DAPI (blue on merge). Bars = 5 µm.

Wheat and barley carry six and two *CENH3* genes, respectively, encoding the α– and βCENH3 variants. Pairwise identity matrixes of the wheat and barley αCENH3 amino acid sequences revealed an identity of 77% while the βCENH3 proteins shared a sequence identity of 69% (**Supplementary Figure 2**). Despite the high similarity scores, multiple sequence alignments identified a short polymorphic region within the N-terminal tail of the CENH3 proteins (**Figure 2A**). The 3D structural models (**Supplementary Figure 3**) indicated that this region is the most disordered and unstructured part of the protein. Peptide sequences differing between the corresponding wheat and barley CENH3 variants were then selected to produce species- and variant-specific polyclonal antibodies (see Materials and methods).

Immunolabelling with the anti-wheat αCENH3 (abbreviation: Wα) and anti-wheat βCENH3 (Wβ) antibodies revealed 22-39 dot-like signals (**Supplementary Figure 4**) in wheat root-tip nuclei (n=15 and n=11, respectively). No signal was observed in barley nuclei when using the two anti-wheat CENH3 antibodies (n=10 and n=11, respectively), demonstrating their species-specificity. Immunolabelling with the anti-barley αCENH3 (Bα) antibody produced 7-14 dot-like fluorescence signals in barley root-tip nuclei (n=19), whilst no signal was detected in wheat nuclei (n=9). The fluorescence signals were organised within one nuclear hemisphere in all cases, in the vicinity of its periphery (**Supplementary Figure 4**), consistently with centromere organisation in the nucleus. The anti-barley βCENH3 (Bβ) antibody showed a very faint or no specific fluorescence signal both in barley (n=18) and wheat (n=12) (**Supplementary Figure 4**) and was thus omitted from further cytological analyses. Based on these results, the Wα, Wβ, and Bα anti-CENH3 antibodies proved to be species-specific while the Bβ anti-CENH3 antibody was not suitable for immunocytochemical assays.

### The anti-wheat and anti-barley CENH3 antibodies co-localise with centromeric repeats in a species-specific manner

To verify the specific binding of the three anti-CENH3 antibodies designed in this study to the core centromeres, we performed immunoFISH by simultaneously labelling the CENH3 proteins and the centromeric DNAs of wheat and barley.

The antibodies designed to detect wheat α– or βCENH3 variants were applied together with the *in situ* hybridisation probe visualising the centromeric retrotransposon of wheat (CRW), an LTR retrotransposon specific to the core centromere. The signals produced by the Wα and Wβ anti-CENH3 antibodies co-localised with the CRW retrotransposon in wheat somatic nuclei (n=18 and n=15, respectively) indicating that the two antibodies detect the centromeric region in wheat (**Figure 2B**). To visualise the barley centromere, the G+C-rich satellite sequence was used as a FISH probe (**Figure 2B**). The Bα CENH3 immunosignal co-localised with the nuclear FISH signal by the G+C repeats in barley somatic nuclei (n=22) demonstrating that the Bα anti-CENH3 antibody recognises the centromeric region in barley (**Figure 2B**).

These results confirmed that the Wα, Wβ, and Bα anti-CENH3 antibodies detect the centromeric regions in the corresponding species and they are thus suitable for further examination of the parental centromere function in wheat × barley hybrids.

### Centromeric CENH3 incorporation in mitotic nuclei of wheat × barley F1 hybrids

**Figure 3:**
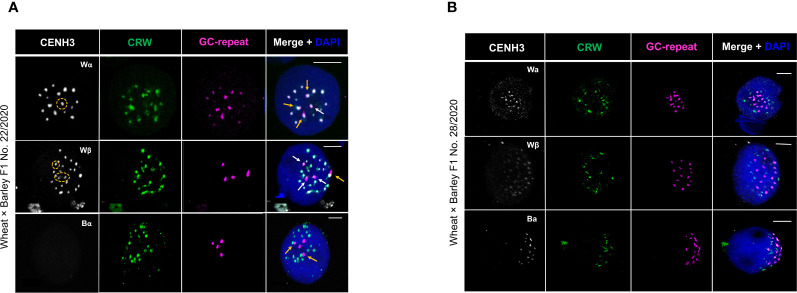
Immunolocalisation of CENH3 proteins in somatic nuclei of wheat × barley hybrid No. 22/2020 **(A)** and No. 28/2020 **(B)**. Active centromeres are immunolabelled with anti-CENH3 antibodies (white) while the centromeric retrotransposon of wheat (CRW, green) and the barley centromere-specific G+C-rich repeats (magenta) are detected by FISH. The chromatin is counterstained with DAPI (blue on merge). Yellow arrows on Merge+DAPI images indicate barley centromeres partially co-localising with wheat centromeres; white arrows show individual barley centromeres not associating with wheat centromeres, yellow circles highlight the corresponding wheat CENH3 signal on the same barley centromeres. Bars = 5 µm.

The loading of wheat and barley CENH3 proteins into the parental centromeres was evaluated in the partial hybrid No. 22/2020, carrying the full haploid chromosome complement of wheat (*n*=3*x*=21) and four chromosomes (3H-6H) of barley (**Figure 1**). The absence of the 1H chromosome (presumably eliminated during the early embryonic cell divisions) implied that the gene encoding the Bα CENH3 protein was lacking. To test whether wheat and barley centromeres have the capacity to incorporate cross-species CENH3 proteins mutually or unilaterally, or they load only their conspecific CENH3, we performed immunoFISH with the species-specific anti-CENH3 antibodies as well as the CRW and G+C probes. We have selectively detected the wheat and barley centromeres but the Bα anti-CENH3 antibody failed to produce immunosignal in the centromeres of the somatic nuclei analysed (n=8; **Figure 3A**), which confirmed the absence of the Bα CENH3 protein in the wheat × barley hybrid No. 22/2020.

ImmunoFISH with the Wα or Wβ anti-CENH3 antibodies and the CRW and G+C probes revealed 11-24 wheat centromeric signals and 2-4 barley centromeric signals (n=31 and n=19, respectively; **Figure 3A**). The number of wheat and barley centromeric signals indicated associations between the wheat centromeres and similar associations between the barley centromeres. In some cases, barley centromeres partially co-localised with wheat centromeres (**Figure 3A**, yellow arrows). Within the cells analysed (n=50) 3.9% of the total number of wheat centromeric signals showed partial co-localisation with a barley centromere, resulting in 0.66 wheat-barley centromere co-localisation per cell (**Supplementary Figure 5**). The Wα and Wβ CENH3 immunosignals co-localised with the CRW sequences of the wheat centromeres pointing to normal maternal centromere activity. Similarly, the signal produced by the Wα and Wβ CENH3 protein antibodies co-localised with the G+C signals of the barley centromeric DNA, irrespective of whether they occurred individually or in association with the wheat centromeres (**Figure 3A**, white and yellow arrows). The presence of wheat CENH3 within the barley centromeres in the wheat × barley hybrid No. 22/2020 indicates a level of plasticity for the wheat CENH3 protein supported by its capacity to follow a cross-species loading fashion and to become incorporated into the barley centromeres.

Cytological analysis of hybrid No. 28/2020 showed 14 barley chromosomes, representing the full diploid genome of barley and a variable number (0-20) of wheat chromosomes (**Figure 1A**: lower panel, **Supplementary Figure 1**). ImmunoFISH in somatic nuclei with Wα, Wβ, and Bα CENH3 immunolabelling and simultaneous *in situ* hybridisation with the CRW and G+C wheat and barley centromere-specific probes (n=24, n=21, and n=16, respectively) revealed a variable number of wheat centromeric signals ranging from 0-19 along with 7-14 barley centromeric signals arranged into one group close to the nuclear periphery (**Figure 3B**). The large variation in the number of wheat centromeric signals and occasionally their complete lack pointed to the progressive elimination of the wheat chromosome set. Only 47.5% of the mitotic cells analysed (n=61) carried wheat centromeres besides the barley centromeres (**Supplementary Table 1**). In 44.8% of these cells, we identified a species-specific organisation, where barley centromeres formed a group at the nuclear periphery, which was surrounded by the wheat centromeres residing on the outskirts of the centromere group (**Figure 3B**, **Supplementary Table 1**). The number of barley centromeric signals corresponded to that counted for the barley somatic nuclei (**Figure 2B**, **Supplementary Figure 4**) revealing that barley centromere-centromere associations take place in the hybrid nuclei as well. The elimination of wheat chromosomes coincided with a less intense or missing Wα CENH3 signal within the wheat centromeres (**Figure 3B**). In contrast, the barley centromeres showed a clear Wα CENH3 signal. The Wβ CENH3 signals also co-localised with both the wheat and barley centromeres and their number ranged from 8 to 22 (**Figure 3B**. ImmunoFISH with the Bα anti-CENH3 antibody revealed incorporation of the CENH3 protein into the barley centromeres but no immunosignal could be detected in the wheat centromeres by our methodology (**Figure 3B**). These observations demonstrated that the wheat CENH3 proteins are incorporated and thus functional in all centromeres of the hybrid nuclei even in the presence of Bα CENH3.

### Centromeric CENH3 incorporation in meiotic nuclei of wheat × barley F1 hybrids

To study the cross-loading of wheat CENH3 into the barley centromeric regions during early meiosis, we performed immunoFISH in meiotic prophase I nuclei of the wheat × barley F1 hybrids No. 22/2020 and 28/2020.

**Figure 4:**
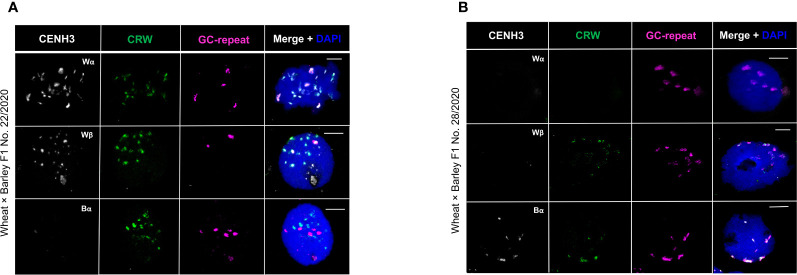
Immunolocalisation of CENH3 proteins in meiotic nuclei of wheat × barley hybrid No. 22/2020 **(A)** and No. 28/2020 **(B)**. Active centromeres are immunolabelled with anti-CENH3 antibodies (white) while the centromeric retrotransposon of wheat (CRW, green) and the barley centromere-specific G+C-rich repeats (magenta) are detected by FISH. The chromatin is counterstained with DAPI (blue on merge). Bars = 5 µm.

In the partial hybrid No. 22/2020, the number of wheat centromeric signals ranged from 11-20 (n=18; **Figure 4A**). The barley centromeric signals marked by the G+C-rich satellite probe ranged from 2-4 (**Figure 4A**), as had been observed in the somatic cells (**Figure 3A**). Our results confirmed that the wheat CENH3 antibody signals co-localised with wheat and barley centromeres in the meiocytes of hybrid No. 22/2020 (**Figure 4A**).

In the meiotic nuclei of hybrid No. 28/2020, only barley centromeres could be detected revealing the complete elimination of the wheat chromosomes (n=16; **Figure 4B**). Accordingly, signals produced by the wheat-specific Wα and Wβ CENH3 antibodies could not be detected in any of the meiotic samples analysed. The number of the barley centromeric signals ranged between 5-13 and Bα CENH3 signal co-localised with the barley centromeres (**Figure 4B**).

## Discussion

### Centromeric CENH3 incorporation in a partial wheat × barley F1 hybrid

We have shown that in F1 hybrid No. 22/2020 carrying a haploid wheat chromosome set and barley chromosomes lacking the 1H encoding αCENH3, wheat CENH3 variants can incorporate into the centromeres of both parental genomes. The retention of the four barley chromosomes and their maintenance through consecutive mitoses in hybrid No. 22/2020 indicated that barley chromosomes can be stably inherited despite the lack of the conspecific CENH3 protein variants (here barley αCENH3) as outlined below.

The *cereba* retroelement is a highly conserved motif within the grass centromeric DNA (Presting et al., 1998; Qi et al., 2013). Individual copies of the barley *cereba* and its wheat CRW orthologue share a sequence homology of ca. 85% (Liu et al., 2008). This high level of homology may, at least partially, account for the successful incorporation of wheat CENH3 protein into the barley centromeres. Although barley centromeres are also interspersed with inherently different repetitive sequences, such as the G+C-rich satellite, the interaction between the centromeric nucleosome and CENH3 proteins within the *Triticeae* tribe is not entirely conservative as CENH3 can be deposited into neocentromeric repeats completely absent from the native centromere (Nasuda et al., 2005). Furthermore, despite the sequence divergence in CENH3 proteins even between closely related species they can be substituted between distant phylogenetic groups. For example, the centromeres of a CENH3 null mutant of *Arabidopsis thaliana* incorporated orthologous CENH3 variants from progressively distant species to complement the lack of the native CENH3 protein (Maheshwari et al., 2015 and Maheshwari et al., 2017).

Analysis of the meiotic nuclei in hybrid No. 22/2020 revealed a similar CENH3-loading pattern to that observed in the somatic nuclei. Two-four barley centromere-specific signals indicated that the four barley chromosomes were still present in the meiotic prophase I of this hybrid. The varying number of wheat centromeric signals observed in meiotic nuclei is consistent with the wheat centromeres undergoing centromere-centromere associations during meiotic prophase I (Sepsi et al., 2017).

### Centromeric CENH3 incorporation in a full wheat × barley F1 hybrid

The gradual elimination of the wheat chromosomes was evident from the cytological examination of the somatic nuclei of hybrid No. 28/2020. Unexpectedly, the full paternal chromosome set (2*n*=2*x*=14) was detected in mitotic metaphase spreads, which may be the result of male meiotic restitution (De Storme and Geelen, 2013). Wheat chromosome elimination coincided with a specific nuclear localisation: the wheat centromeres surrounded the barley centromeres that were organised into one group at the nuclear periphery. While wheat αCENH3 was present in barley centromeres, only a poor intensity staining could be observed in the exteriorised wheat chromosomes. This was in agreement with data on *H. vulgare* × *H. bulbosum* hybrids where paternal chromosome elimination coincided with peripheral centromere compartmentalisation and the loss of CENH3 (Sanei et al., 2011). This observation is widely supported by the known peripheral pattern of spatial localisation of the parental chromosome set destined to be eliminated in mitoses (Schwarzacher et al., 1992; Kim et al., 2002; Mochida et al., 2004) and even interphases (Gernand et al., 2005) in various interspecific cereal hybrids. CENH3 unloading and nuclear chromosome exteriorisation may thus be a conserved strategy for genome elimination within the *Triticeae* tribe.

In the somatic nuclei of hybrid No. 28/2020, the wheat CENH3 proteins were incorporated into the wheat and barley centromeres. Barley αCENH3, however, was only detected in the barley centromeres and was absent in the wheat centromeres, which may be unique to the specific plant material and caused by progressive epigenetic chromosome silencing leading to CENH3 unloading and subsequent chromosome elimination. The capacity of the stable barley centromeres to incorporate both wheat and barley CENH3 provides important evidence that co-loading CENH3 proteins from the two parental species into the same centromere maintains chromosome stability at mitosis. This is contrary to data obtained with taxonomically more remote organisms: CENH3 from a species as distant as *Zea mays* could complement (mitotically and meiotically) the *Arabidopsis* CENH3 null mutant. However, when complemented plants were crossed to the wild type, the hybrid progeny exhibited extensive mis-segregation, aneuploidy, and fertility loss (Maheshwari et al., 2015). Mis-segregation involved chromosomes with diverse CENH3s, which suggests that although the essential function of CENH3 may be conserved across distant species, co-loading diverse CENH3s into the same centromere may weaken centromeres, leading to genome elimination. Our study showed that incorporating wheat and barley CENH3 proteins within the same centromeric regions (by loading both wheat CENH3 and at least barley αCENH3) did not affect the maintenance of the duplicated barley genome. In contrast, the coexistence of the diverse CENH3 proteins within the same nucleus coincided with the apparent instability of the wheat genome.

By the onset of meiosis within the anthers of hybrid No. 28/2020 the wheat chromosome set became eliminated. Conversely, the strong anti-barley αCENH3 antibody immunosignals in the barley centromeres suggested normal expression of barley *CENH3* genes. Due to the presence of the full diploid genome, the double copy number of the barley chromosome set in the somatic cells may lead to higher quantities of barley CENH3 proteins vs. wheat CENH3s, which together with the flexibility of the barley centromere to incorporate both conspecific and wheat CENH3 proteins, rendered barley centromeres dominant over those of wheat in hybrid No. 28/2020.

### Patterns of CENH3 incorporation in interspecific cereal hybrids

**Figure 5:**
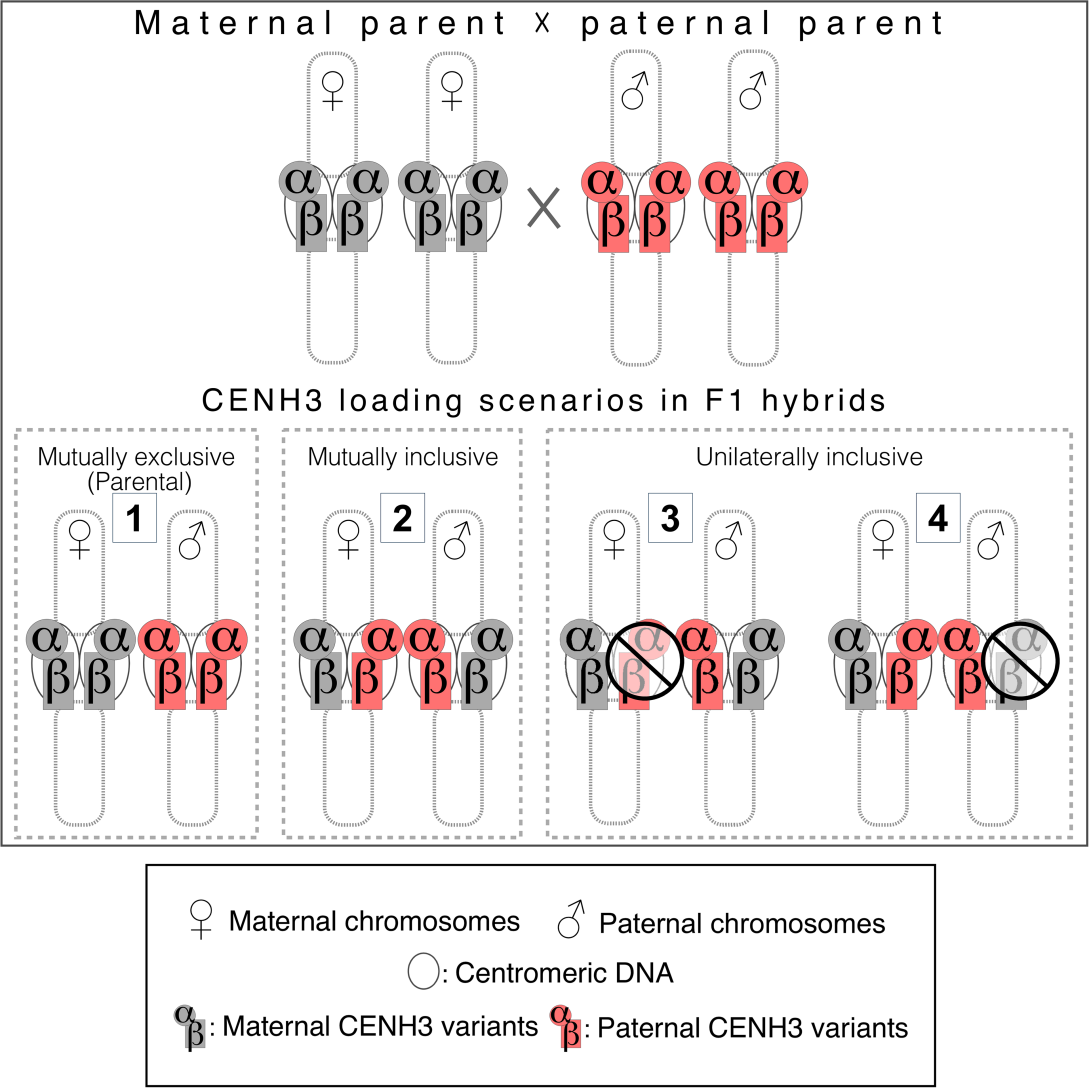
Schematic presentation of potential scenarios for CENH3 incorporation into the centromeres of interspecific F1 hybrids.

In principle, the modes of CENH3 protein incorporation into the centromeres in interspecific hybrids can take one or more of the following paths (**Figure 5**): mutually exclusive, i.e., preserving the parental loading pattern (No. 1 in the figure), mutually inclusive in both parents (permitting cross-specific CENH3 incorporation, No. 2) or unilaterally inclusive (only either of the two parents incorporates cross-species CENH3, Nos. 3-4). These scenarios may be specific and therefore need to be evaluated for each of the CENH3 variants in the presence or absence of their counterpart variant(s) in both somatic and meiotic nuclei.

Our data point to the presence of scenario No. 3 in the interspecific combination of wheat × barley, i.e., the unilateral inclusivity (plasticity) of the barley centromere to incorporate the two maternal (wheat) CENH3 variants in the absence of barley αCENH3 and in the presence of the two barley CENH3 variants too, although evidence for the latter could only be obtained in somatic nuclei.

With these scenarios in mind, we analysed published experimental data and evaluated them in the context of cereals. Based on immunolabelling as direct evidence for CENH3 incorporation, the data were distributed in three groups according to the type of material used: (i) primary hybrids, (ii) established aneuploid (chromosome additions) or euploid (substitutions, translocations) genetic stocks, and (iii) transgenically produced alien CENH3 combinations.

In the two interspecific hybrid combinations tested so far, *Hordeum vulgare* × *H. bulbosum* (Sanei et al., 2011) and oat × pearl millet (Ishii et al., 2015b), the maternal CENH3 was incorporated into all the paternal centromeres in somatic nuclei. In barley, both *H. vulgare* CENH3 variants effectively occupied the *H. bulbosum* centromeres in the presence as well as in the absence of the conspecific CENH3 proteins.

Stable genetic stocks can be viewed as end-products mirroring the prior chromosome elimination process. Four derivative types from interspecific crosses have so far been studied by CENH3 immunolabelling of somatic nuclei, metaphase chromosomes, and chromatin fibres: (i) a disomic 7H chromosome substitution of *H. bulbosum* in barley (Sanei et al., 2011), (ii) disomic additions of maize chromosome 3 (Wang et al., 2014) and chromosome 6 (Jin et al., 2004) to oat, (iii) disomic substitutions of *Thinopyrum elongatum* (Guo et al., 2016) and *Th. intermedium* (Li et al., 2023) chromosomes in wheat. Finally, (iv) several wheat-rye lines were also tested: monosomic 2R and 6R chromosome addition lines (Guo et al., 2016), over 100 1RS.1BL translocation lines containing hybrid wheat-rye centromeres (Wang et al., 2017), and similar hybrid centromeres in a reconstructed wheat 1B chromosome (Karimi-Ashtiyani et al., 2021). In all these cases, a clear maternal (barley, oat, and wheat) CENH3 incorporation was observed in the individual paternal centromeres either in the presence (Jin et al., 2004) or in the absence of their conspecific *CENH3* genes. The only exception to this scenario was the double-disomic 1H+6H barley chromosome additions to wheat: here all four *CENH3* genes were expressed but besides the two wheat variants (scenario No. 3) barley αCENH3 (but not βCENH3) was also incorporated in all centromeres (Sanei et al., 2011). These data present overwhelming evidence for the predominance of scenario No. 3 (**Figure 5**), which points to the plasticity of the paternal centromere to incorporate cross-species CENH3 proteins over a wide range of interspecific hybrids in the *Triticeae* tribe.

The cases of transgenic and native CENH3 combinations cannot be interpreted according to the listed scenarios because of the absence of parental relations. These reconstructed situations are suitable for establishing the boundaries of cross-species CENH3 loading in a homogeneous genetic background rather than for testing cross-specific incorporation into the centromeres in a hybrid genome. In addition, transgenic CENH3 proteins are usually detected indirectly via a large fluorescent protein tag, which is known to interfere with native CENH3 activity (Kalitsis et al., 2003; Ravi et al., 2011; Britt and Kuppu, 2016). It is therefore of no surprise that YFP-tagged maize CENH3 was not detected in the centromeres of transgenic wheat (Chen et al., 2015) or wheat-maize somatic hybrids (Yang et al., 2019).

The α– and β*CENH3* genes are the result of a gene duplication event, dated back to 35-40 million years ago (about the divergence time of the *Pooideae* subfamily from the *Oryzoideae* and *Panicoideae*), which modified the exon-intron structure of the original *CENH3* gene (Elisafenko et al., 2021). At this time scale, specialisation and subfunctionalisation could have occurred between the CENH3 paralogues as demonstrated in cowpea about generative development (Ishii et al., 2020). The similar incorporation of the two wheat CENH3 proteins into the barley centromeres in somatic and meiotic nuclei of the two wheat × barley hybrids points to no such functional deviation between the paralogous proteins as also observed in the non-hybrid background of barley (Ishii et al., 2015a) and rye (Evtushenko et al., 2021).

## Conclusion

We have demonstrated the plasticity of barley centromere to incorporate wheat CENH3 proteins in interspecific wheat × barley F1 hybrids. This is another new example of the centromere plasticity and CENH3-centromere interaction in the two most important crop species within the *Triticeae* tribe. While in previous cases the phenomenon of plasticity was attributed to rapid centromere expansion and restructuring at the intraspecific level (Schneider et al., 2016; Ma et al., 2023) as well as in stable hybrids (Zhao et al., 2019), here it is apparently associated with the native centromere diversity (without obvious induced amplification) naturally available in newly formed interspecific hybrids.

Further analysis of epigenetic and genetic features of centromeres within a wider range of cereal species is needed to understand and influence chromosome stability and elimination in crop improvement programmes. Utilising the driving force of the accelerated evolution rate of centromeric DNA and CENH3 proteins would be instrumental in advanced plant breeding, allowing the production of hybrid combinations so far inaccessible for crop improvement.

## Data availability statement

The original contributions presented in the study are included in the article/**Supplementary Material**. Further inquiries can be directed to the corresponding authors.

## Author contributions

EM: Conceptualization, Investigation, Writing – review & editing. DP: Investigation, Writing – review & editing. AL-T: Investigation, Writing – review & editing. DM: Investigation, Writing – review & editing. AF: Data curation, Investigation, Visualization, Writing – review & editing. MA: Investigation, Writing – review & editing. AK: Formal analysis, Investigation, Visualization, Writing – review & editing. AS: Conceptualization, Supervision, Visualization, Writing – original draft, Writing – review & editing. LS: Conceptualization, Formal analysis, Funding acquisition, Project administration, Supervision, Validation, Writing – original draft, Writing – review & editing.
