# Supplementary material for: Plasticity of parental CENH3 incorporation into the centromeres in wheat × barley F1 hybrids
Source: Front Plant Sci. 2024 Jan 19;15:1324817. doi: 10.3389/fpls.2024.1324817 (PMC10834757; doi:10.3389/fpls.2024.1324817)
Supplement: Supplementary file 1 [file Presentation_1.pptx]

## Slide 1
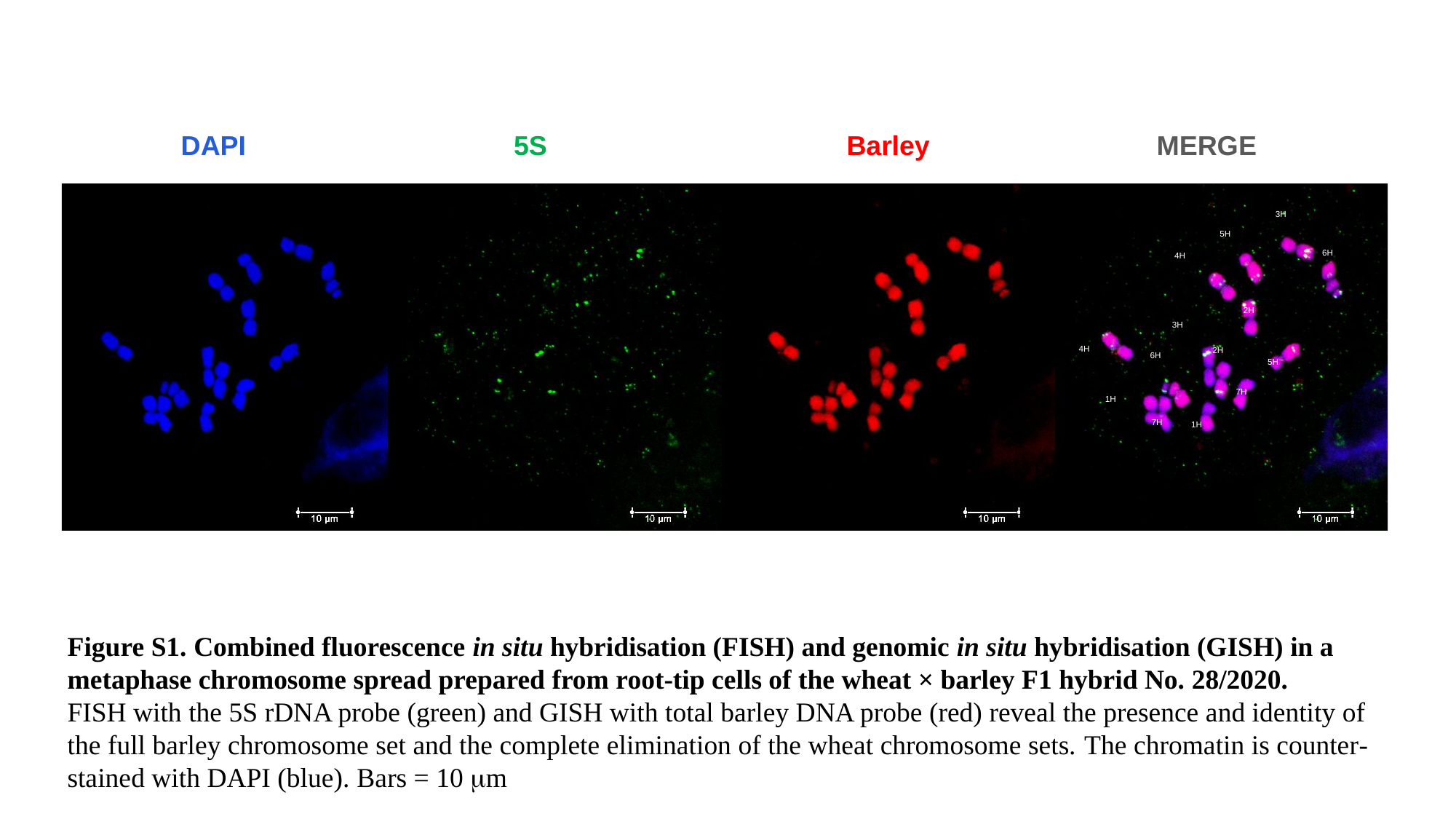

DAPI
5S
Barley
MERGE
3H
5H
6H
4H
2H
3H
4H
2H
6H
5H
7H
1H
7H
1H
Figure S1. Combined fluorescence in situ hybridisation (FISH) and genomic in situ hybridisation (GISH) in a metaphase chromosome spread prepared from root-tip cells of the wheat × barley F1 hybrid No. 28/2020.
FISH with the 5S rDNA probe (green) and GISH with total barley DNA probe (red) reveal the presence and identity of the full barley chromosome set and the complete elimination of the wheat chromosome sets. The chromatin is counter-stained with DAPI (blue). Bars = 10 mm

## Slide 2
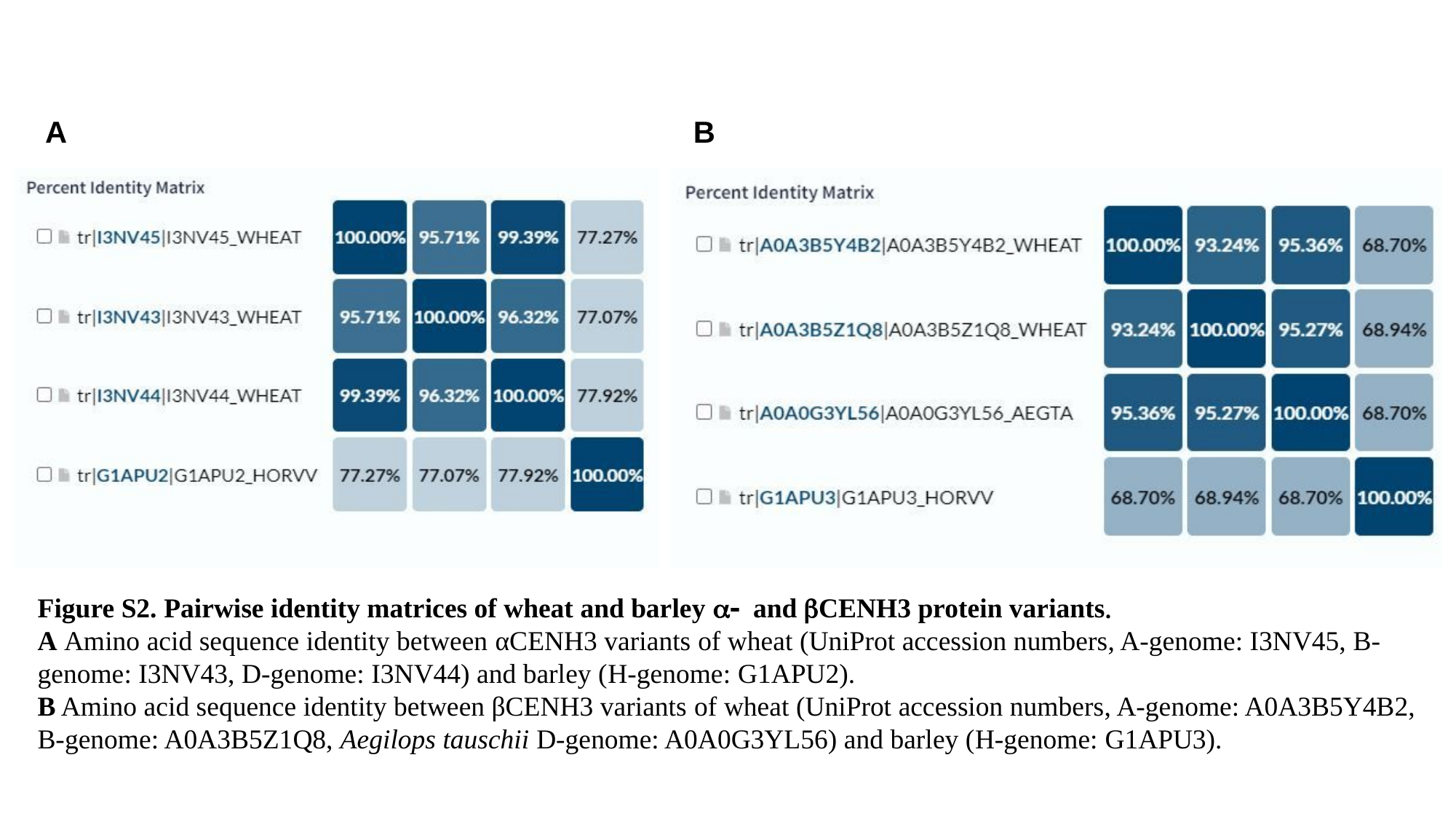

A
B
Figure S2. Pairwise identity matrices of wheat and barley a- and bCENH3 protein variants.
A Amino acid sequence identity between αCENH3 variants of wheat (UniProt accession numbers, A-genome: I3NV45, B-genome: I3NV43, D-genome: I3NV44) and barley (H-genome: G1APU2).
B Amino acid sequence identity between βCENH3 variants of wheat (UniProt accession numbers, A-genome: A0A3B5Y4B2, B-genome: A0A3B5Z1Q8, Aegilops tauschii D-genome: A0A0G3YL56) and barley (H-genome: G1APU3).

## Slide 3
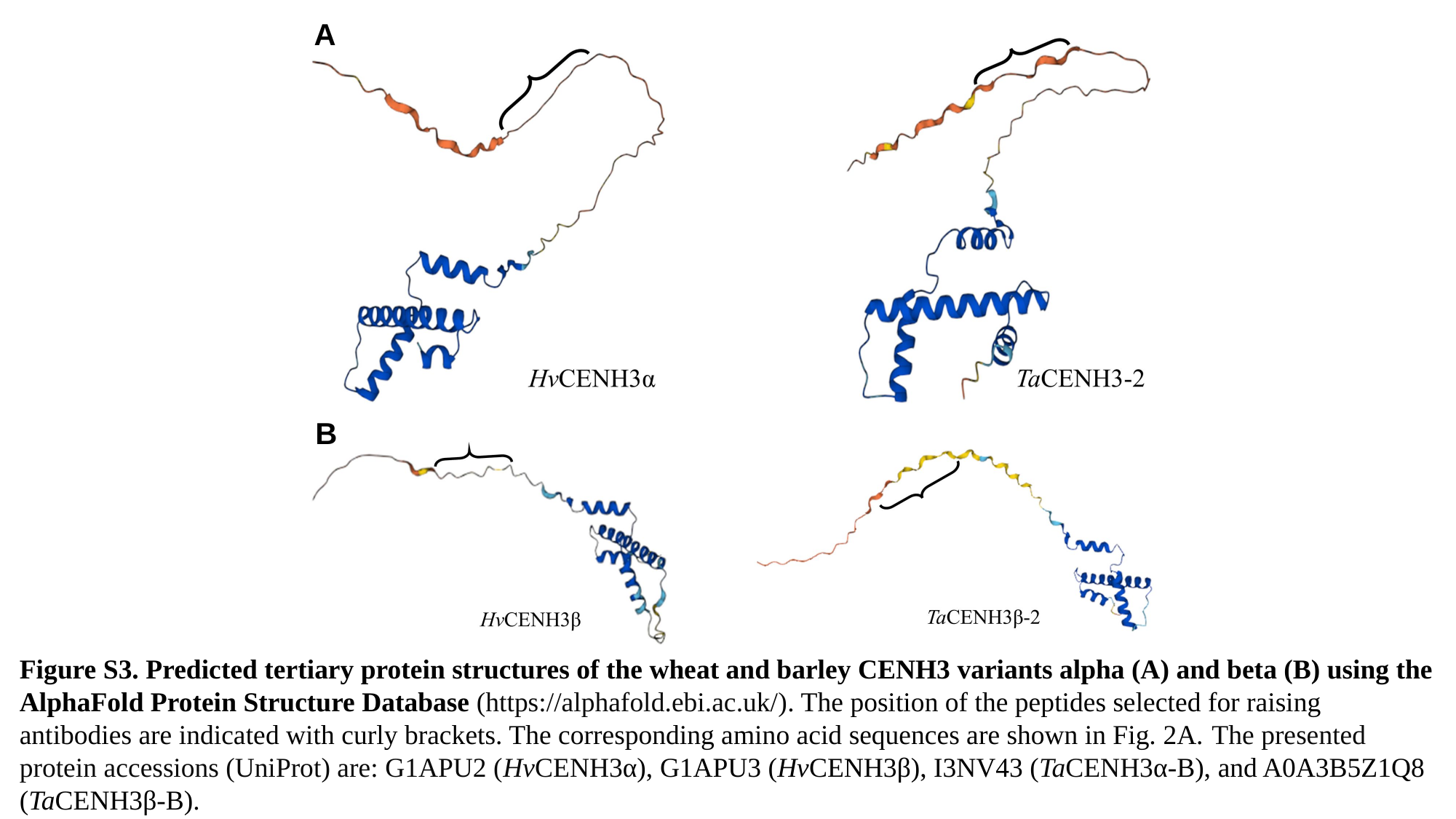

A
B
Figure S3. Predicted tertiary protein structures of the wheat and barley CENH3 variants alpha (A) and beta (B) using the AlphaFold Protein Structure Database (https://alphafold.ebi.ac.uk/). The position of the peptides selected for raising antibodies are indicated with curly brackets. The corresponding amino acid sequences are shown in Fig. 2A. The presented protein accessions (UniProt) are: G1APU2 (HvCENH3α), G1APU3 (HvCENH3β), I3NV43 (TaCENH3α-B), and A0A3B5Z1Q8 (TaCENH3β-B).

## Slide 4
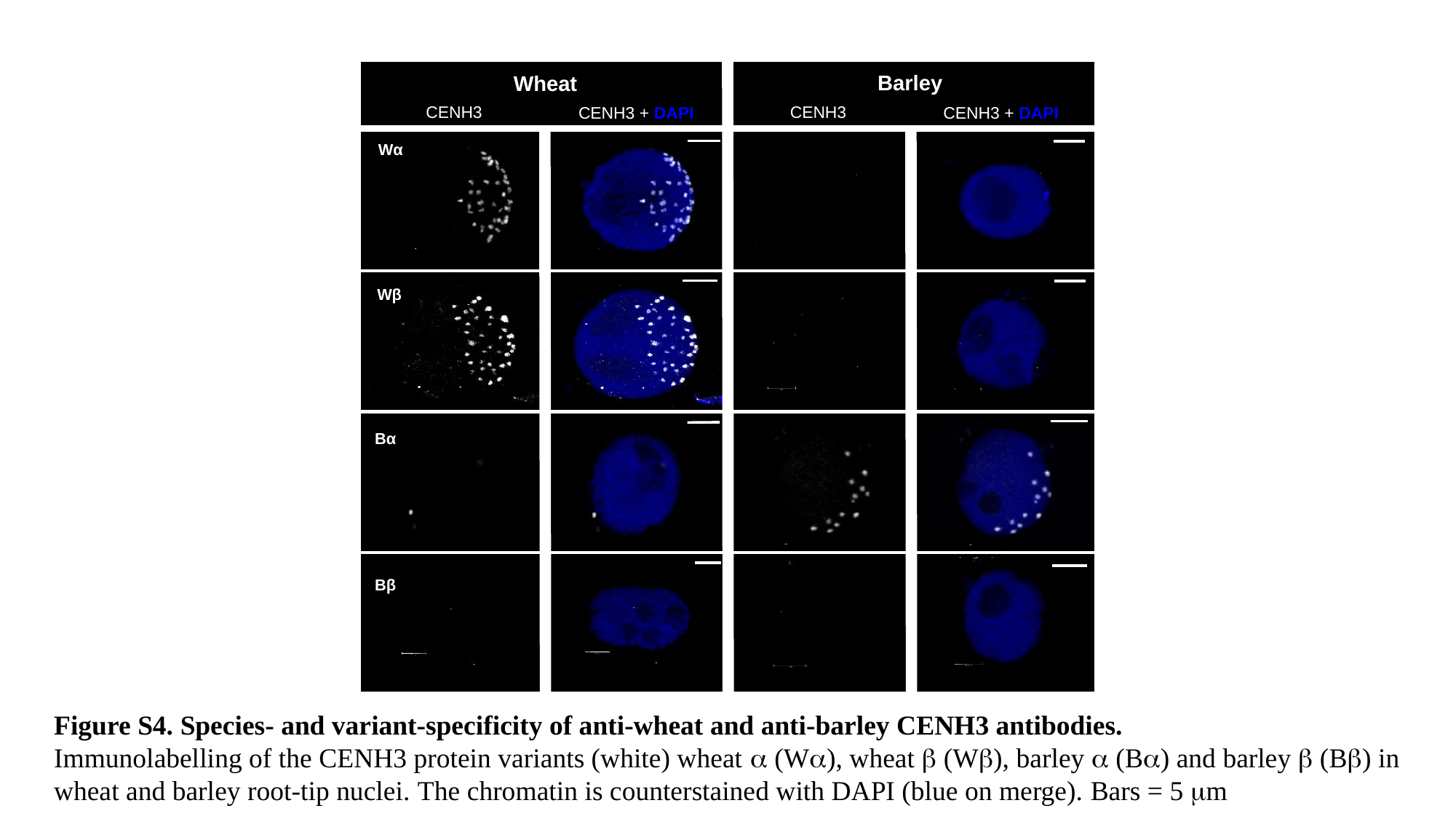

Barley
Wheat
CENH3
CENH3
CENH3 + DAPI
CENH3 + DAPI
Wα
 Wβ
 Bα
 Bβ
Figure S4. Species- and variant-specificity of anti-wheat and anti-barley CENH3 antibodies.
Immunolabelling of the CENH3 protein variants (white) wheat a (Wa), wheat b (Wb), barley a (Ba) and barley b (Bb) in wheat and barley root-tip nuclei. The chromatin is counterstained with DAPI (blue on merge). Bars = 5 mm

## Slide 5
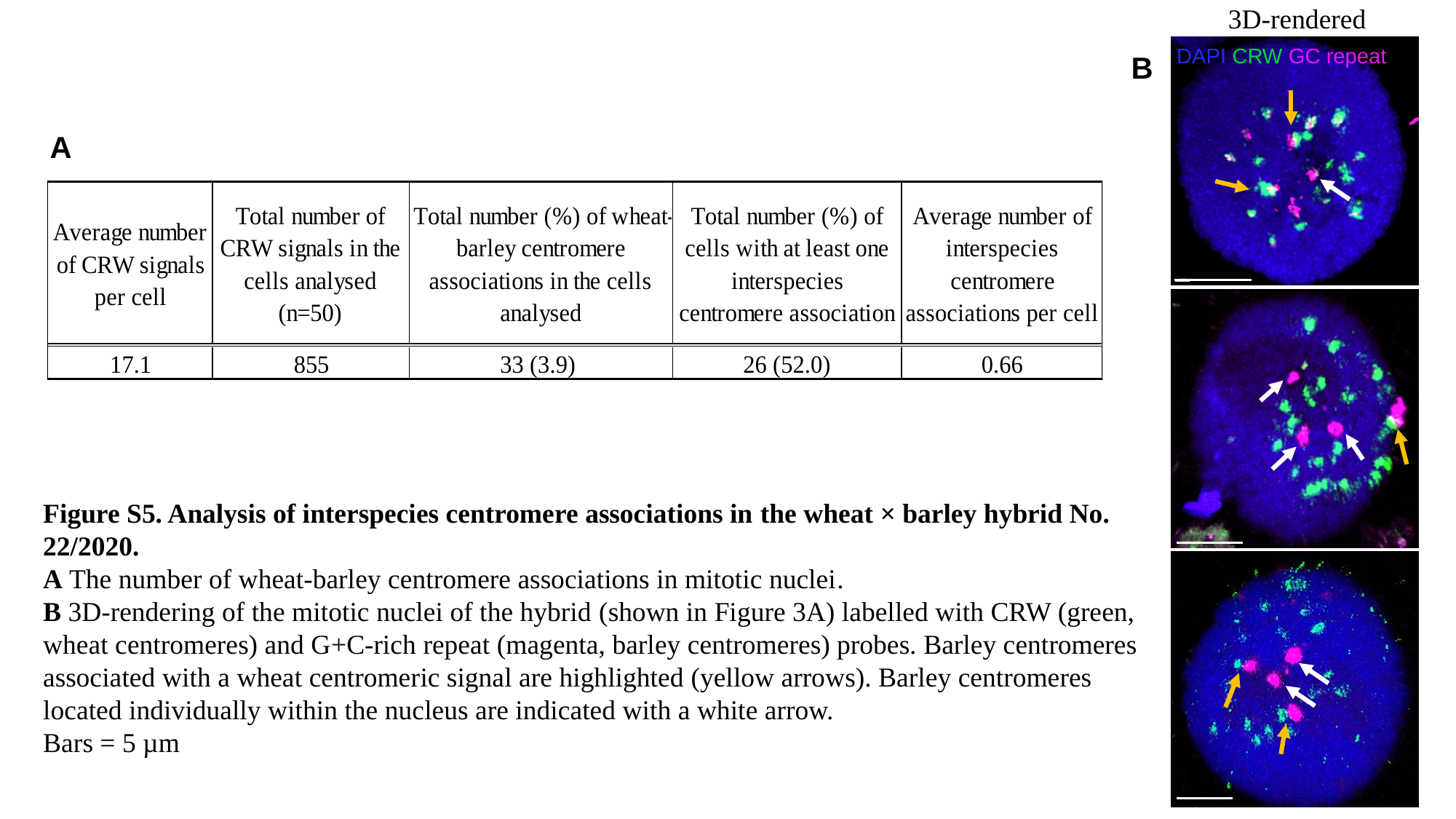

3D-rendered
DAPI CRW GC repeat
B
A
5um
Figure S5. Analysis of interspecies centromere associations in the wheat × barley hybrid No. 22/2020.
A The number of wheat-barley centromere associations in mitotic nuclei.
B 3D-rendering of the mitotic nuclei of the hybrid (shown in Figure 3A) labelled with CRW (green, wheat centromeres) and G+C-rich repeat (magenta, barley centromeres) probes. Barley centromeres associated with a wheat centromeric signal are highlighted (yellow arrows). Barley centromeres located individually within the nucleus are indicated with a white arrow.
Bars = 5 µm

## Slide 6
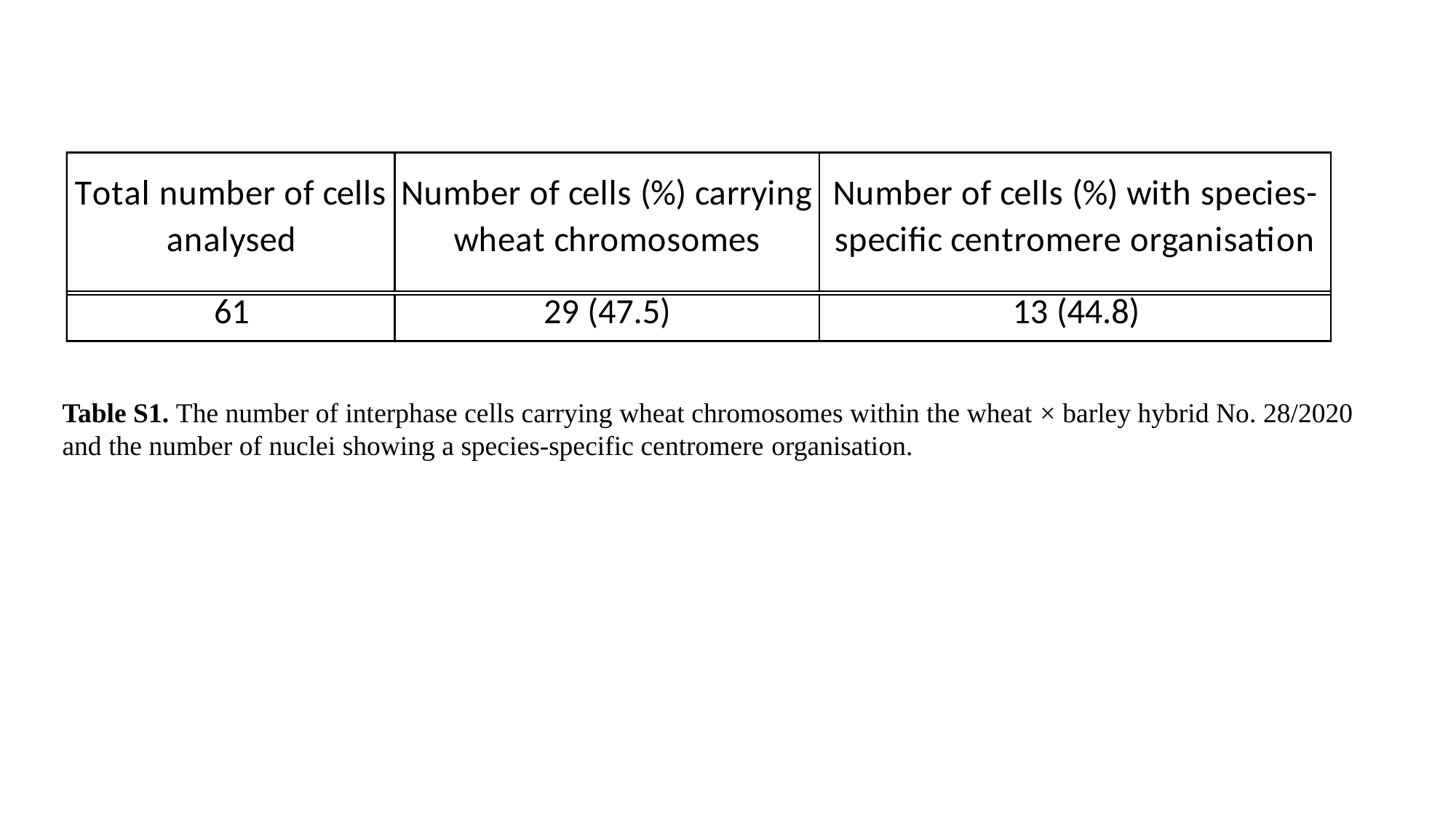

Table S1. The number of interphase cells carrying wheat chromosomes within the wheat × barley hybrid No. 28/2020 and the number of nuclei showing a species-specific centromere organisation.
